# FAIRSCAPE: An Evolving AI-readiness Framework for Biomedical Research

**DOI:** 10.1101/2024.12.23.629818

**Published:** 2025-01-05

**Authors:** Sadnan Al Manir, Maxwell Adam Levinson, Justin Niestroy, Christopher Churas, Jillian A. Parker, Timothy Clark

**Affiliations:** 1 University of Virginia School of Medicine; 2 University of California San Diego School of Medicine; 3 University of Virginia School of Data Science

## Abstract

**Motivation:**

Artificial intelligence (AI) applications require explainability (XAI) for FAIR, ethical deployment, whether in the clinic or in the laboratory. Richly descriptive XAI metadata representing how pre-model data were obtained, characterized, transformed, and distributed, should be available along with the data prior to training and application of AI models.

**Results:**

The FAIRSCAPE framework generates, packages, and integrates critical pre-model XAI descriptive metadata, including deep provenance graphs and data dictionaries with feature validation on uploaded data, software, and computations, with special reference to biomedical datasets. It provides ethical and semantic characterization of the dataset along with licensing and availability information, and integrates seamlessly with NIH-recommended generalist repositories. The server is cloud-compliant and implemented in Python3. Client software in Python3 is callable from the command line or directly as python functions. We provide a REST API, and a GUI-based client in javascript is also available.

**Availability and implementation:**

The code is freely available under MIT license and is hosted at https://fairscape.github.io/, along with comprehensive documentation and tutorials.

## 1. Introduction

Artificial intelligence (AI) readiness is a critical acceptance factor for biomedical datasets intended for use in ethical, FAIR (Findable, Accessible, Interoperable, Reusable)^1^ AI applications, whether in the clinic or the laboratory. At the conclusion of an AI analysis, we must be able to explain and interpret the results. This requires full transparency, including complete pre-model characterization of the input data and its various transformations, from patient or laboratory instrument to model training and execution^2^.

The FAIRSCAPE framework was developed to provide such transparency. It was initially developed for applications in critical care medicine at the University of Virginia Center for Advanced Medical Analytics^3^, and later extended and re-implemented to encompass functional genomics^4^ and clinical applications in the NIH-funded Bridge2AI program. We continue to develop its capabilities to track the AI-readiness requirements of the program^5^, as specified by the Bridge2AI Standards Working Group, representing investigators from forty leading institutions participating in the program.

FAIRSCAPE is intended to aid in the establishment and support of pre-model AI explainability^6^ through capturing rich information about biomedical datasets and software components involved in extracting and computing those datasets. The system creates deep metadata including provenance graphs and dataset schemas, providing FAIR-compliant persistent identifiers (PIDs) for biomedical data and software. It supports a simple direct upload process to any instance of the Harvard Dataverse^7^, an NIH-approved generalist repository and member of the NIH Generalist Repository Ecosystem Initiative (GREI). Interfaces to other GREI repositories are planned for the near-term.

**Figure 1.**
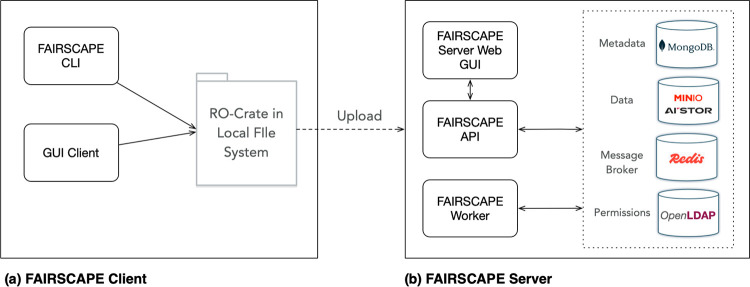
Architecture of the FAIRSCAPE framework. (a) The FAIRSCAPE CLI and GUI Client tools annotate, package, and validate data into FAIRSCAPE-compliant RO-Crates. (b) The FAIRSCAPE Server API accepts incoming ROCrates, then extracts, processes and validates metadata, assigning ARK Persistent IDs for every ROCrate, Software, Dataset, and Computation Object. Datasets are then stored and retrieved from Minio AIStor Object Storage. Metadata for all objects is stored in MongoDB for querying. User permissions and API keys are stored in Open LDAP.

An architectural sketch of the FAIRSCAPE framework is shown in Figure 1. An example set of commands to build an RO-Crate for a very simple table of subject data with BMI computation and output dataset, with the output RO-Crate, and a visualization of its JSON-LD provenance graph, is given in Supplemental Data.

FAIRSCAPE is a powerful and robust tool for supporting AI-readiness, specifically adapted for use with biomedical datasets. *It currently supports twenty-two of the twenty-eight AI-readiness criteria recommended by the NIH Bridge2AI Program for FAIR, ethically sourced and managed biomedical datasets*^5^.

We plan to extend the framework to meet the remaining six criteria with additional functionality and metadata in the coming year.

FAIRSCAPE is an open-source product licensed under permissive MIT license.

## 2. Software Implementation

FAIRSCAPE comprises a set of Python packages consisting of two different client-side versions and a server that together create, manage, upload, archive, and export Research Object (RO)-Crate packages^8^ with detailed provenance graphs based on the Evidence Graph Ontology (EVI)^9^. It also supports extending the metadata package, which is serialized in JSON-LD.

The FAIRSCAPE-CLI client is pip-installable and may be called either from the command line or directly as Python functions. It builds an RO-Crate and incrementally adds its components in a set of JSON-LD graphs using the pattern:

Computation <uses> Software) ∧
Input <usedBy> Computation <generates> Output


Each component includes additional associated descriptive metadata that employs the schema.org vocabulary. Users may extend the metadata they associate with RO-Crates. FAIRSCAPE-CLI uses the Frictionless Data framework^10^ to generate JSON Schema definitions for tabular and HDF5 files, which are associated with their referenced datasets. Validation utilizing Frictionless ensures that datasets conform to their provided schemas. Every RO-Crate component receives a locally unique key. Data may be directly packaged, or simply referenced using a URI. Once an RO-Crate is packaged it may be uploaded directly to the server, where the local keys become resolvable ARK persistent IDs.

FAIRSCAPE also supports a GUI client based on electron, React, and javascript. The tool walks the user through RO-Crate initialization and component upload. At each step, it displays a form to collect the required metadata, with the resulting JSON-LD metadata shown on the side of the application. After completing all the required forms, users can review their created RO-Crate and its contents, package it into a zip file, and upload it to a FAIRSCAPE instance.

The FAIRSCAPE server, also in Python, receives, catalogs, indexes, and stores uploaded RO-Crate zip packages, extracts and registers their components and associated metadata, and stores that information. The server API uses the FastAPI framework and provides REST API access. All digital objects, from the zip files to any included datasets or software not included by reference to another repository, are managed in an S3-compliant database (e.g., in the case discussed here, MinIO) or any cloud-based object store with an S3 API. Metadata is managed in the Mongo noSQL database.

The server leverages the REDIS in-memory cache as a message broker, to pass information and commands from the API to the internal Worker process for execution. Multi-user and group permissioning is handled using OpenLDAP, an open-source authorization system. OpenLDAP stores user credentials, permissions, and encrypted tokens.

Objects stored in FAIRSCAPE may be pushed directly to any instance of the Dataverse academic repository system, provided the user has previously acquired and stored a Dataverse token in their FAIRSCAPE account.

## 3. Results

### Server Installation:

- Pull the latest server code, compose YAML from git, and set working directory

git clone http://github.com/fairscape/mds_python
cd mds_python
- Build the FAIRSCAPE services

docker compose up --build

### Server Configuration:

Important server environment variables are noted here: https://fairscape.github.io/getting-started/configuration/

### CLI Client Installation & Test:

- Install: pip install fairscape-cli
- Test: fairscape-cli --help
  Note: Python 3.8 or higher is a requirement for this package.

### GUI Client Installation:

- Go to https://github.com/fairscape/FairscapeGUIClient/releases
- Download and run the latest version for your operating system (Windows, macOS, or Linux).
  Note: On macOS, users may need to open the application from the Finder window.

### Client Functions

- The FAIRSCAPE-CLI command line client’s functions are documented here: https://pypi.org/project/fairscape-cli/
- The FAIRSCAPE GUI Client functions are documented here: https://fairscape.github.io/fairscape_gui_client/instructions/

### Server REST API Calls

- The FAIRSCAPE server’s REST API documentation is generated from FastAPI by SWAGGER, available here: https://fairscape.net/api/docs

### Server Web GUI

The FAIRSCAPE Server provides a Web GUI interface to view metadata and download RO-Crates uploaded to FAIRSCAPE. After logging in, a dashboard displays all of the researcher’s uploaded RO-Crates, with links to download them or visit their landing page. Each landing page presents multiple views of an RO-Crate:

- a table summary of its required JSON-LD metadata and the files it contains,
- serializations of its metadata in JSON-LD, RDF, and Turtle, and
- a d3 visualization of the evidence graph, built upon the jsonld-vis package, for exploring its provenance.

Users can publish their RO-Crates to any Dataverse instance through a form accessible from its landing page. While an RO-Crate’s metadata is publicly available, file downloads and RO-Crate publishing are restricted to project members. The Server Web GUI provides visual access to all FAIRSCAPE API endpoints, enabling researchers to list, view, download, and publish their data.

An RO-Crate produced by the calls described above is attached as Supplementary data.

## 4. Conclusions

The FAIRSCAPE AI-readiness Framework provides a common platform for creating, managing, and distributing ethical, FAIR, AI-ready biomedical datasets. The initial version was developed at the Center for Advanced Medical Analytics at the University of Virginia. The current version was developed to support datasets produced in the NIH Bridge2AI program and is under continuous development in line with recommendations of the Bridge2AI Standards Working Group, while continuing to support clinical research programs at the UVA School of Medicine.

Near-term development objectives for 2025 include integration with the Portable Encapsulated Projects (PEP) genomics framework, support for the Bridge2AI Critical Care Medicine Grand Challenge (CHoRUS), support for statistical characterization of input datasets, and expansion of metadata to include Bridge2AI Model Cards and Datasheets.

We welcome communications from users outside this program, and are open to further collaborations to extend the framework.
