# Supplementary material for: FAIRSCAPE: An Evolving AI-readiness Framework for Biomedical Research
Source: bioRxiv. 2024 Dec 23:2024.12.23.629818. Preprint. [Version 1] doi: 10.1101/2024.12.23.629818 (PMC11703166; doi:10.1101/2024.12.23.629818)

Supplemental Data for

FAIRSCAPE: An Evolving AI-Readiness Framework for Biomedical Research

We provide supplemental data below to show how an RO-Crate is created and handled by FAIRSCAPE, with display of its package contents:

1. Input commands to produce an RO-Crate dataset package for a calculation;
2. Directory listing for an RO-Crate produced by these commands;
3. JSON-LD from that RO-Crate's `ro-crate-metadata.json` file;
4. Python code from `bmi_calculator.py` software referenced in the RO-Crate;
5. Input dataset;
6. Output dataset;
7. Graphical view of the EVI provenance graph produced by the FAIRSCAPE Server landing page for this RO-Crate package.

Metadata for this computation tells us the source of the data, the creators of the software and the dataset, how the data is structured, the actual computation that was run, and the resulting output file.

1. Input Commands

```
fairscape-cli rocrate create \
    --name "BMI Calculation Example" \
    --author "Last F., Second A." \
    --description "Example RO-Crate demonstrating BMI calculation from
height and weight data" \
    --organization-name "Example Org" \
    --project-name "Health Metrics" \
    --keywords "health" \
    --keywords "bmi" \
    "./bmi_rocrate"

fairscape-cli schema infer \
    --name "Health Measurements Schema" \
    --description "Schema for health measurements including height and
weight" \
    ./health_measurements.csv \
    ./infer_health_measurements_schema.json

fairscape-cli schema infer \
    --name "Health Measurements with BMI Schema" \
    --description "Schema for health measurements including height, weight
and calculated BMI" \
    ./health_measurements_with_bmi.csv \
    ./infer_health_measurements_bmi_schema.json

fairscape-cli rocrate add dataset \
    --name "Health Measurements" \
    --author "Last F., Second A." \
```

```
    --version "1.0" \
    --date-published "2024-12-19" \
    --description "Sample dataset containing height and weight
measurements" \
    --keywords "health" \
    --data-format "CSV" \
    --schema "ark:59852/schema-health-measurements-schema-20241219-170933"
\
    --source-filepath "./health_measurements.csv" \
    --destination-filepath "./bmi_rocrate/health_measurements.csv" \
    "./bmi_rocrate"

fairscape-cli rocrate add software \
    --name "BMI Calculator" \
    --author "Last F., Second A." \
    --version "1.0" \
    --description "Python script to calculate BMI from height and weight
measurements" \
    --keywords "health" \
    --keywords "bmi" \
    --file-format "py" \
    --source-filepath "./bmi_calculator.py" \
    --destination-filepath "./bmi_rocrate/bmi_calculator.py" \
    --date-modified "2024-12-19" \
    "./bmi_rocrate"

fairscape-cli rocrate add dataset \
    --name "Health Measurements with BMI" \
    --author "Last F., Second A." \
    --version "1.0" \
    --date-published "2024-12-19" \
    --description "Dataset containing height, weight measurements and
calculated BMI" \
    --keywords "health" \
    --keywords "bmi" \
    --data-format "CSV" \
    --schema "ark:59852/schema-health-measurements-with-bmi-schema-
20241219-171016" \
    --derived-from "ark:59852/dataset-health-measurements-W7domj80Ao" \
    --source-filepath "./health_measurements_with_bmi.csv" \
    --destination-filepath
"./bmi_rocrate/health_measurements_with_bmi.csv" \
    "./bmi_rocrate"

fairscape-cli rocrate register computation \
    --name "BMI Calculation Process" \
    --run-by "Last F., Second A." \
    --date-created "2024-12-19" \
```

```
    --description "Process to calculate BMI from height and weight
measurements" \
    --keywords "health" \
    --keywords "bmi" \
    --used-software "ark:59852/software-bmi-calculator-oPNG9gOld8k" \
    --used-dataset "ark:59852/dataset-health-measurements-W7domj80Ao" \
    --generated "ark:59852/dataset-health-measurements-with-bmi-
vlifjeUmBK" \
    "./bmi_rocrate"
```

2. RO-Crate Package Contents

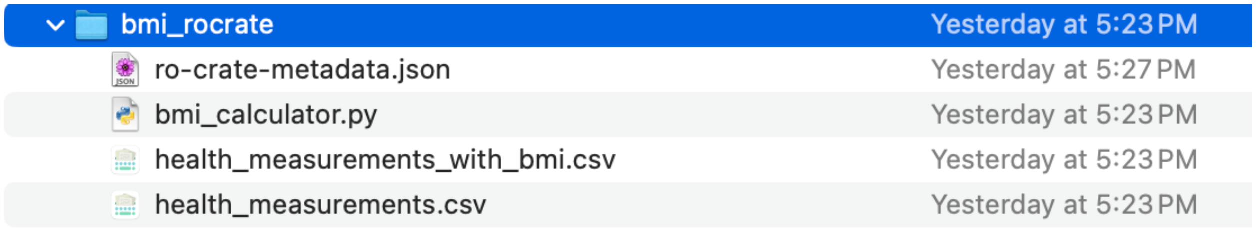

3. RO-Crate Metadata from the `ro-crate-metadata.json file`


```
{
  "@id": "ark:59852/rocrate-bmi-calculation-example-GkKZXaPUbW",
  "@type": "https://w3id.org/EVI#ROCrate",
  "context": {
    "@vocab": "https://schema.org/",
    "EVI": "https://w3id.org/EVI#"
  },
  "name": "BMI Calculation Example",
  "author": "Last F., Second A.",
  "description": "Example RO-Crate demonstrating BMI calculation from
height and weight data",
  "keywords": ["health", "bmi"],
  "isPartOf": [
    {
      "@id": "ark:59852/organization-example-org-s6g7TxB8GT3",
      "@type": "Organization",
      "name": "Example Org"
    },
    {
      "@id": "ark:59852/project-health-metrics-qT1mIhQ3Y3M",
      "@type": "Project",
      "name": "Health Metrics"
    }
  ],
  "@graph": [
    {
      "@id": "ark:59852/dataset-health-measurements-W7domj80Ao",
      "@context": {
```

```
        "@vocab": "https://schema.org/",
        "evi": "https://w3id.org/EVI#"
      },
      "@type": "https://w3id.org/EVI#Dataset",
      "name": "Health Measurements",
      "keywords": ["health", "anthropometry"],
      "description": "Sample dataset containing height and weight
measurements",
      "author": "Last F., Second A.",
      "datePublished": "2024-12-19",
      "version": "1.0",
      "format": "CSV",
      "schema": "ark:59852/schema-health-measurements-schema-20241219-
170933",
      "generatedBy": [],
      "derivedFrom": [],
      "usedBy": [],
      "contentUrl": "file:///health_measurements.csv"
    },
    {
      "@id": "ark:59852/software-bmi-calculator-oPNG9gOld8k",
      "@context": {
        "@vocab": "https://schema.org/",
        "evi": "https://w3id.org/EVI#"
      },
      "@type": "https://w2id.org/EVI#Software",
      "name": "BMI Calculator",
      "keywords": ["health", "bmi"],
      "description": "Python script to calculate BMI from height and
weight measurements",
      "author": "Last F., Second A.",
      "dateModified": "2024-12-19",
      "version": "1.0",
      "format": "py",
      "usedByComputation": [],
      "contentUrl": "file:///bmi_calculator.py"
    },
    {
      "@id": "ark:59852/dataset-health-measurements-with-bmi-vlifjeUmBK",
      "@context": {
        "@vocab": "https://schema.org/",
        "evi": "https://w3id.org/EVI#"
      },
      "@type": "https://w3id.org/EVI#Dataset",
      "name": "Health Measurements with BMI",
      "keywords": ["health", "bmi"],
      "description": "Dataset containing height, weight measurements and
calculated BMI",
      "author": "Last F., Second A.",
```

```
      "datePublished": "2024-12-19",
      "version": "1.0",
      "format": "CSV",
      "schema": "ark:59852/schema-health-measurements-with-bmi-schema-
20241219-171016",
      "generatedBy": [],
      "derivedFrom": ["ark:59852/dataset-health-measurements-W7domj80Ao"],
      "usedBy": [],
      "contentUrl": "file:///health_measurements_with_bmi.csv"
    },
    {
      "@id": "ark:59852/computation-bmi-calculation-process-6XbJ4NygehY",
      "@context": {
        "@vocab": "https://schema.org/",
        "evi": "https://w3id.org/EVI#"
      },
      "@type": "https://w3id.org/EVI#Computation",
      "name": "BMI Calculation Process",
      "keywords": ["health", "bmi"],
      "description": "Process to calculate BMI from height and weight
measurements",
      "runBy": "Last F., Second A.",
      "dateCreated": "2024-12-19",
      "usedSoftware": ["ark:59852/software-bmi-calculator-oPNG9gOld8k"],
      "usedDataset": ["ark:59852/dataset-health-measurements-W7domj80Ao"],
      "generated": ["ark:59852/dataset-health-measurements-with-bmi-
vlifjeUmBK"]
    },
    {
      "@id": "ark:59852/schema-health-measurements-schema-20241219-
170933",
      "@context": {
        "@vocab": "https://schema.org/",
        "EVI": "https://w3id.org/EVI#"
      },
      "@type": "EVI:Schema",
      "$schema": "https://json-schema.org/draft/2020-12/schema",
      "name": "Health Measurements Schema",
      "description": "Schema for health measurements including height and
weight",
      "type": "object",
      "separator": ",",
      "header": true,
      "required": ["person_id", "height_cm", "weight_kg"],
      "properties": {
        "person_id": {
          "description": "Unique identifier for each person",
          "index": 0,
          "type": "string",
```

```
          "pattern": "^P[0-9]{3}$"
        },
        "height_cm": {
          "description": "Height measurement in centimeters",
          "index": 1,
          "type": "number"
        },
        "weight_kg": {
          "description": "Weight measurement in kilograms",
          "index": 2,
          "type": "number"
        }
      },
      "additionalProperties": true
    },
    {
      "@id": "ark:59852/schema-health-measurements-with-bmi-schema-
20241219-171016",
      "@context": {
        "@vocab": "https://schema.org/",
        "EVI": "https://w3id.org/EVI#"
      },
      "@type": "EVI:Schema",
      "$schema": "https://json-schema.org/draft/2020-12/schema",
      "name": "Health Measurements with BMI Schema",
      "description": "Schema for health measurements including height,
weight and calculated BMI",
      "type": "object",
      "separator": ",",
      "header": true,
      "required": ["person_id", "height_cm", "weight_kg", "bmi"],
      "properties": {
        "person_id": {
          "description": "Unique identifier for each person",
          "index": 0,
          "type": "string",
          "pattern": "^P[0-9]{3}$"
        },
        "height_cm": {
          "description": "Height measurement in centimeters",
          "index": 1,
          "type": "number"
        },
        "weight_kg": {
          "description": "Weight measurement in kilograms",
          "index": 2,
          "type": "number"
        },
        "bmi": {
```

```
          "description": "Body Mass Index calculated from height and
weight",
          "index": 3,
          "type": "number"
        }
      },
      "additionalProperties": true
    }
  ]
}
```


4. Python code from `bmi_calculator.py` as packged in the RO-Crate;

```
import pandas as pd

def calculate_bmi(height_cm, weight_kg):
    height_m = height_cm / 100
    return (weight_kg / (height_m * height_m)).round(1)

def process_health_data(input_file, output_file):
    df = pd.read_csv(input_file)
    df['bmi'] = calculate_bmi(df['height_cm'], df['weight_kg'])
    df.to_csv(output_file, index=False)

if __name__ == "__main__":
    process_health_data('health_measurements.csv',
'health_measurements_with_bmi.csv')
```

5. Input Dataset Contents of `health_measurements.csv`

person_id	height_cm	weight_kg
P001	175	92
P002	168.6	66.6
P003	176.5	71
P004	185.2	48.6
P005	167.7	61.8
P006	167.7	71.7
P007	185.8	52.7
P008	177.7	75.6
P009	165.3	61
P010	175.4	65.6
P011	165.4	61
P012	165.3	97.8
P013	172.4	69.8
P014	150.9	54.1
P015	152.8	82.3
P016	164.4	51.7
P017	159.9	73.1

P018	173.1	40.6
P019	160.9	50.1
P020	155.9	73

6. Output Dataset Contents of `health_measurements_with_bmi.csv`

person_id	height_cm	weight_kg	bmi
P001	175	92	30
P002	168.6	66.6	23.4
P003	176.5	71	22.8
P004	185.2	48.6	14.2
P005	167.7	61.8	22
P006	167.7	71.7	25.5
P007	185.8	52.7	15.3
P008	177.7	75.6	23.9
P009	165.3	61	22.3
P010	175.4	65.6	21.3
P011	165.4	61	22.3
P012	165.3	97.8	35.8
P013	172.4	69.8	23.5
P014	150.9	54.1	23.8
P015	152.8	82.3	35.2
P016	164.4	51.7	19.1
P017	159.9	73.1	28.6
P018	173.1	40.6	13.5
P019	160.9	50.1	19.4
P020	155.9	73	30

7. Provenance Graph for the Result Dataset

Graphical display of provenance graph as provided by the FAIRSCAPE Web GUI's landing page for this RO-Crate.

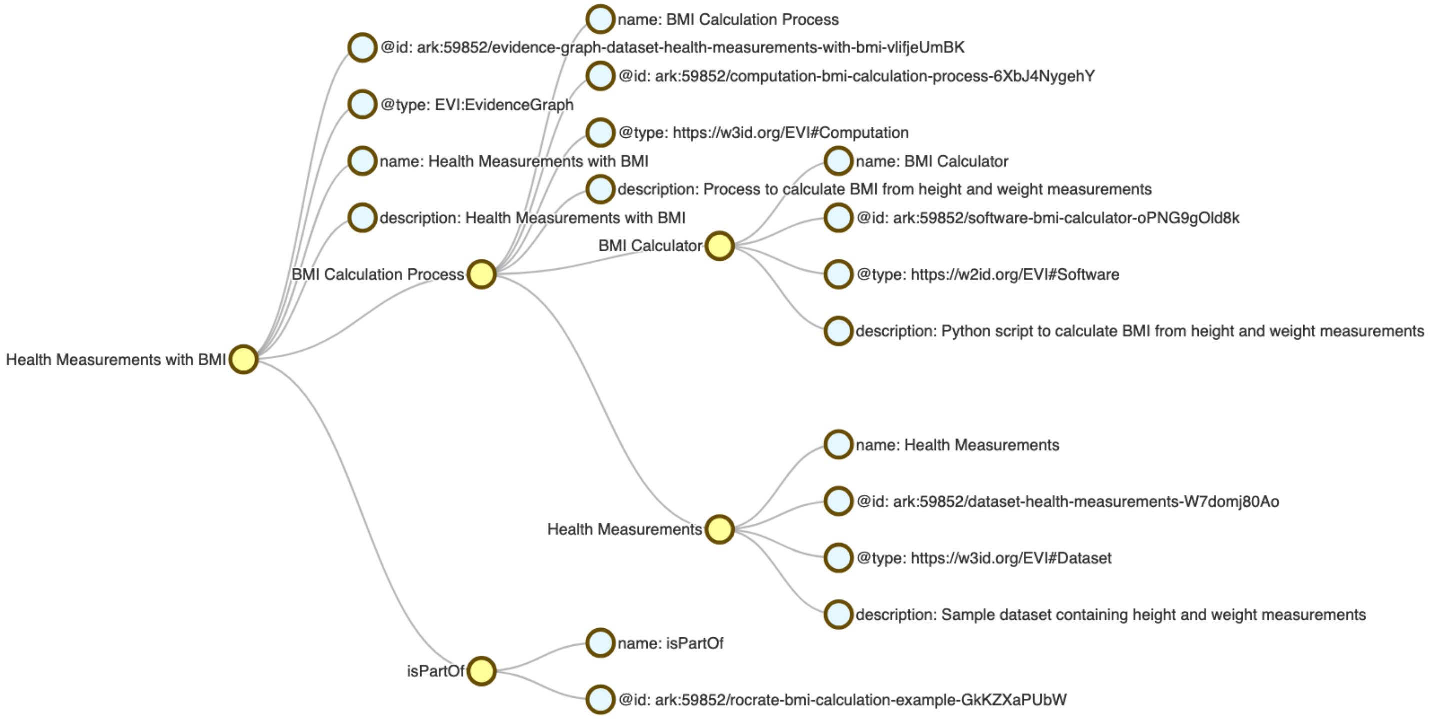

Supplement: Supplementary file 1 [file media-1.pdf]
